# New algorithms for unsupervised cell clustering from scRNA-seq data

**DOI:** 10.1093/bioadv/vbag121

**Published:** 2026-04-29

**Authors:** Melissa Robles, Jorge Díaz-Riaño, Cristhian Forigua, Sebastian Ojeda, Laura Guio, Paula Siaucho, Jennifer Guzman-Porras, Danilo García-Orjuela, Andres Naranjo, Silvia Maradei, Adolfo Quiroz, Jorge Duitama

**Affiliations:** Systems and Computing Engineering Department, Universidad de los Andes, Bogotá, Colombia; Systems and Computing Engineering Department, Universidad de los Andes, Bogotá, Colombia; Systems and Computing Engineering Department, Universidad de los Andes, Bogotá, Colombia; Systems and Computing Engineering Department, Universidad de los Andes, Bogotá, Colombia; HOMI, Fundación Hospital Pediátrico La Misericordia, Bogotá, Colombia; Biotecnología y Genética SAS, Biotecgen, Bogotá, Colombia; HOMI, Fundación Hospital Pediátrico La Misericordia, Bogotá, Colombia; Biotecnología y Genética SAS, Biotecgen, Bogotá, Colombia; HOMI, Fundación Hospital Pediátrico La Misericordia, Bogotá, Colombia; Biotecnología y Genética SAS, Biotecgen, Bogotá, Colombia; Department of Mathematics, Universidad de los Andes, Bogotá, Colombia; Systems and Computing Engineering Department, Universidad de los Andes, Bogotá, Colombia

## Abstract

The identification of cell types is a basic step of pipelines for Single-Cell RNA sequencing (scRNA-seq) data analysis. However, unsupervised clustering of cells from scRNA-seq data has multiple challenges: high dimensionality, sparseness of the expression matrix, and technical noise that generates false zero entries. In this study, we introduce new algorithms for clustering scRNA-seq data. The first algorithm builds a *k*-MST graph from distances obtained directly from the input data without dimensionality reduction. The computation follows an iterative procedure of k steps, calculating the edges of minimum spanning trees over different subgraphs obtained by removing edges selected in previous iterations. The Louvain algorithm is executed on the *k*-MST graph for cell clustering. We also explored an alternative based on neural networks, using an autoencoder to learn the parameters of a Gaussian mixture model. Benchmark experiments show that the algorithms have competitive accuracy, compared to previous solutions. Sequencing depth, number of cells and tissue types have important effects on the performance of the algorithms. Further experiments with scRNA-data taken from a patient with refractory epilepsy show that the autoencoder model achieved the best accuracy for this dataset, and the *k*-MST was competitive among graph-based approaches.

## Introduction

The Single Cell RNA sequencing (scRNA-seq) methodology enables the quantification of gene expression at a single cell level. Since the initial proof of concept in 2009 (Tang *et al.* 2009) the use of this technology has increased steadily, thanks to different improvements in cell sorting and library preparation techniques, and the reduction of sequencing costs (Chang *et al.* 2024). Gene expression profiles based on scRNA-seq data can be used to differentiate according to cell type, which refers to the category or classification of cells based on their specific characteristics, such as molecular composition, morphology, or function (Zeng 2022). Aggregating the data by cell types facilitates the identification of genes whose expression significantly differs among the various cell types present in a tissue. Additionally, it improves the identification of genes whose expression varies between tissues affected by diseases and healthy tissues, compared to bulk RNA-seq data.

Identifying cell types is a primary step of the data analysis pipeline for Single-Cell sequencing experiments. However, unsupervised clustering of scRNA-seq data has multiple challenges. One challenge is the high-dimensional nature of the data, where the number of features (genes in this case) can be in the thousands. A large number of features tends to homogenize the distances between samples, which can reduce the accuracy of different clustering algorithms (Giraud 2021). Another challenge is the sparse nature of the gene expression matrix. Not all genes are active in every cell, leading to many zero entries, often representing more than 90% of the data to analyze. The final challenge is the presence of technical noise that can introduce false zero entries, known as “dropouts”.

Multiple methodologies have been proposed for cell type detection in scRNA-seq data. A combination of classical techniques for unsupervised clustering is implemented in the SC3 library (Kiselev *et al.* 2017). SC3 starts with the computation of three distance matrices: Euclidean distance, Spearman correlation, and Pearson correlation, followed by dimensionality reduction of these matrices through principal component analysis (PCA). Subsequently, *k*-means clustering is applied to the three resulting matrices, and finally, hierarchical clustering is performed using the consensus of the three obtained results. Some methodologies, such as SINCERA (Guo *et al.* 2015) and DP-SIMLR (Kausar *et al.* 2019), are based on hierarchical and density clustering algorithms.

Graph-based approaches are also used as cell clustering methodologies for scRNA data. An example of this approach is the SSNN-Louvain methodology (Zhu *et al.* 2020), which relies on community detection with a modified Louvain algorithm on the Shared-Nearest-Neighbor (SNN) graph. A graph-based approach is also implemented in the widely used tool Seurat (Hao *et al.* 2021). This software employs community detection from the graph of *k* nearest neighbors (*k*-NN) of the data obtained after dimensionality reduction using PCA. Although various methods can be applied to obtain the communities, the default approach of Seurat is the Louvain algorithm (Blondel *et al.* 2008). This algorithm optimizes a modularity function, looking for dense connections between nodes within communities, and sparse connections between nodes of different communities. The SLM algorithm (Waltman and van Eck 2013) is an alternative technique to optimize the modularity, available in Seurat. In contrast to the Louvain algorithm, SLM allows the movement of entire sets of nodes and the splitting of communities, providing greater flexibility in exploring solutions for modularity optimization. Constructing the graph itself is particularly crucial when using community detection methods in graphs. Seurat constructs the initial graph from the *k* nearest neighbors graph based on Euclidean distance and defines the weights of the resulting edges as the Jaccard similarity.

Neural networks have also been proposed as alternatives for analysis of scRNA data. In particular, an autoencoder is a type of neural network that can serve to a dual purpose of error correction and dimensionality reduction (Kramer 1991). The models learn to encode data into a lower-dimensional space and then reconstruct it by calculating a loss function that penalizes the differences between the original and reconstructed data. For scRNA-seq data, the use of autoencoders have shown positive results in both data clustering (Eraslan *et al.* 2019; Tian *et al.* 2019) and data imputation (Talwar *et al.* 2018; Chen *et al.* 2023). Following the approach proposed by Tian *et al.* (2021), the encoder architecture can be modified to enable clustering during training, allowing it to learn the parameters of clusters distributions.

In this article, we introduce two novel clustering algorithms for scRNA-seq data. The first algorithm constructs a *k* minimum spanning tree (*k*-MST) graph using pairwise Pearson correlation between cells. Unlike other methods (Hao *et al.* 2021; Kiselev *et al.* 2017), it omits PCA dimensionality reduction and instead selects the genes with the highest upward deviation from variance, adjusted by the mean of the counts. Following this, the Louvain algorithm is applied to identify clusters in the data. The second approach utilizes deep learning, where cluster parameters are learned in a secondary training stage with a GMM-log likelihood loss function. We assessed the accuracy of these methods against previous approaches using both simulated and real datasets, including a novel dataset taken from a patient with refractory epilepsy.

## Algorithms

### Clustering based on the *k*-MST graph

**Figure 1:**
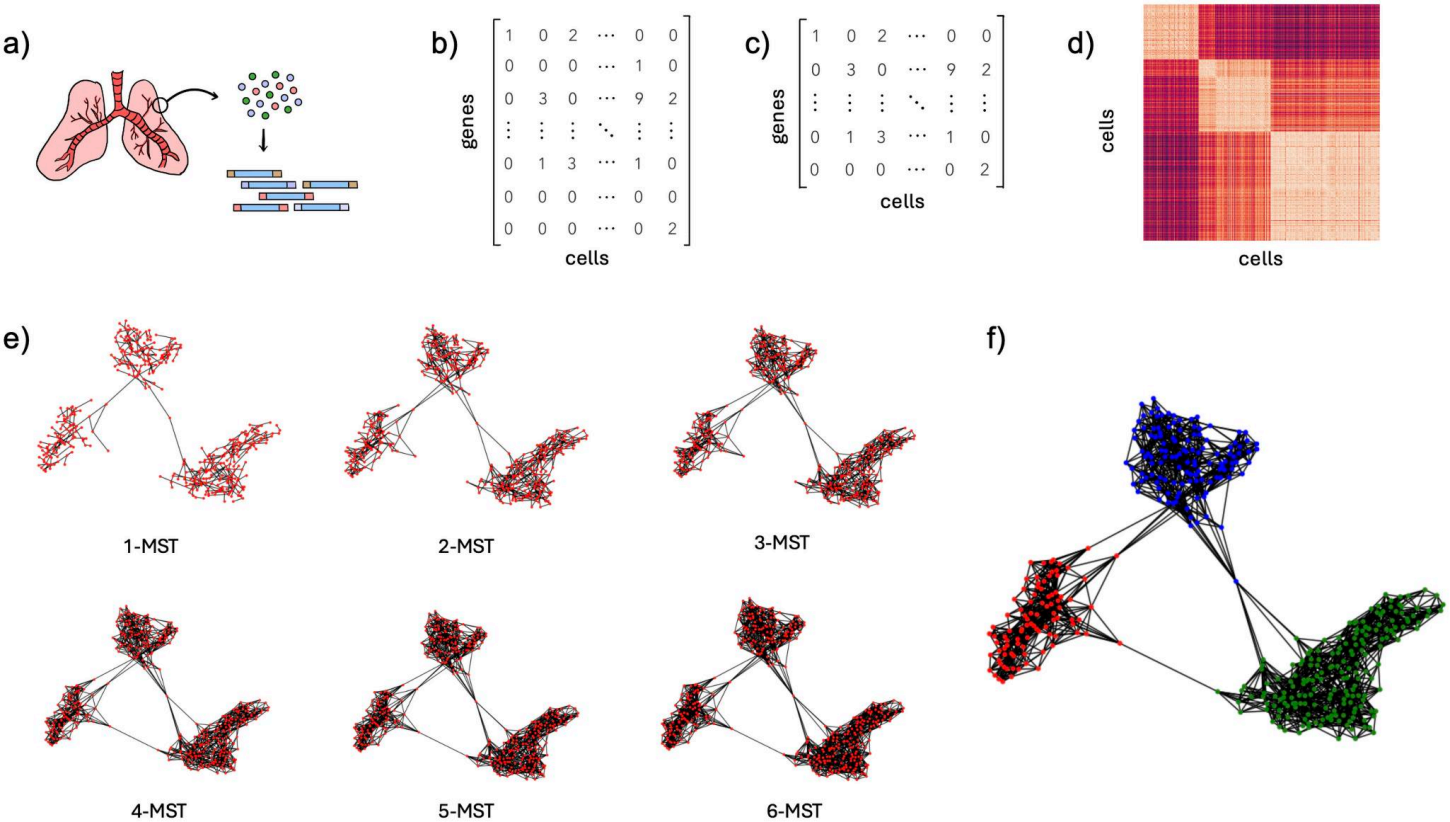
Graph-based scRNA-seq clustering pipeline. (a) Perform single-cell RNA sequencing. (b) Generate the count matrix. (c) Gene filtering: Retain only the 5000 genes with the highest upward deviation from the adjusted variance. (d) Compute the pairwise Pearson correlation between cells. (e) Compute the k-MST graph (f) Apply the Louvain algorithm for community detection.

Figure 1 shows the main steps of the proposed *k*-MST algorithm for analysis of scRNA data. Reads obtained by the experiment (Figure 1a) are processed using one of the standard pipelines to generate the input count matrix, which contains the expression levels of each gene across all cells in the experiment (Figure 1b). During the preprocessing step, genes and samples are filtered using statistics on the expression levels per gene and per cell (Figure 1c, See methods “Preprocessing of the count matrices” for details). Starting from the filtered matrix, the algorithm computes the Pearson’s correlation function over each pair of cells, building a similarity score matrix of dimensions n×n with *n* representing the number of cells in the dataset (Figure 1d): for each pair of gene expression vectors $x_{i}$ and $x_{j}$ corresponding to two different cells, the correlation is defined as:


$$c_{i,j}=\frac{\mathrm{cov}(x_{i},x_{j})}{\sigma_{x_{i}}\sigma_{x_{j}}},$$

Where $\mathrm{cov}(x_{i},x_{j})$ denotes the covariance between $x_{i}$ and $x_{j}$, and $\sigma_{x_{i}}$ and $\sigma_{x_{j}}$ are the standard deviations of $x_{i}$ and $x_{j}$, respectively. The similarity matrix is the input of the main step of the algorithm, which is the construction of the *k*-MST graph (Figure 1e). This graph was first introduced in the context of the multivariate two-sample problem by Friedman and Rafsky in 1979 (Friedman and Rafsky 1979). The computation follows an iterative procedure. Initially, the MST of the original graph is calculated and their edges are selected and removed from the original graph. A second MST is calculated on the resulting graph and the edges are selected and removed again. This iterative process continues for k=log(n) iterations, and the final graph is the union of the *k* “orthogonal” MST graphs. Compared to the k-nearest neighbor (KNN) graph, implemented in tools such as Seurat (Hao *et al.* 2021), the *k*-MST graph is more dense but still ensures that each node has degree at least k.

The Louvain algorithm (Blondel *et al.* 2008) is executed on the resulting graph to partition the nodes, with the goal of optimizing the modularity of a graph *G* and a partition *C* defined as


$$M(G,C)=\sum_{i,j,C(i)=C(j)}\frac{e_{ij}}{T}-\frac{w^{\mathrm{out}}(i)w^{in}(j)}{T^{2}},$$

where the value $e_{ij}$ corresponds to the weight of the edge (i,j), *T* represents the sum of all weight in the graph, $w^{\mathrm{out}}(i)=\sum_{k}e_{ik}$ and $w^{in}(j)=\sum_{k}e_{kj}$.

The Louvain algorithm begins by assigning each node to its own community. Through a series of iterations, nodes are reassigned to different communities to maximize modularity. This process continues until no further improvement in modularity can be achieved, resulting in the identification of stable community structures within the network (Figure 1f).

The time complexity depends on the number of cells *n*, and the number of selected genes *g*. The computation of the pairwise Pearson correlation matrix requires $O(n^{2}g)$ time. However, this step is highly parallelizable and is computed efficiently in practice using optimized linear algebra routines. For the *k*-MST construction, each iteration applies Kruskal’s algorithm over a graph with $O(n^{2})$ edges, resulting in a cost of $O(n^{2}\;\text{log}\;n)$ per iteration. Since the algorithm performs k=log(n) iterations, the total cost of the *k*-MST algorithm is therefore $O(n^{2}g+n^{2}{\;\text{log}\;}^{2}n)$. In practice, the *k*-MST construction is the dominant step.

### Clustering based on deep learning

Building on recent work using autoencoder-based deep learning methods for dimensionality reduction and further clustering, we implemented a two-stage model that integrates count modeling and probabilistic clustering. In the first stage, the autoencoder is pretrained with the zero-inflated negative binomial loss, initially proposed by the developers of the DCA method (Eraslan *et al.* 2019).


LZINB=∑ij−log(ZINB(Xij πij,μij,θij)),

where


ZINB(X=k π,μ,θ)={π+(1−π)·NB(k μ,θ)  if k=0(1−π)·NB(x μ,θ)  if  k>0.

This distribution accounts for the variability in sampling of RNA transcripts and for the excess of zeros introduced by dropout events in single-cell sequencing. In this equation, $\pi_{ij}$ models the probability of dropout, while $\mu_{ij}$ and $\theta_{ij}$ control the mean and dispersion of the negative binomial component, respectively.

**Figure 2:**
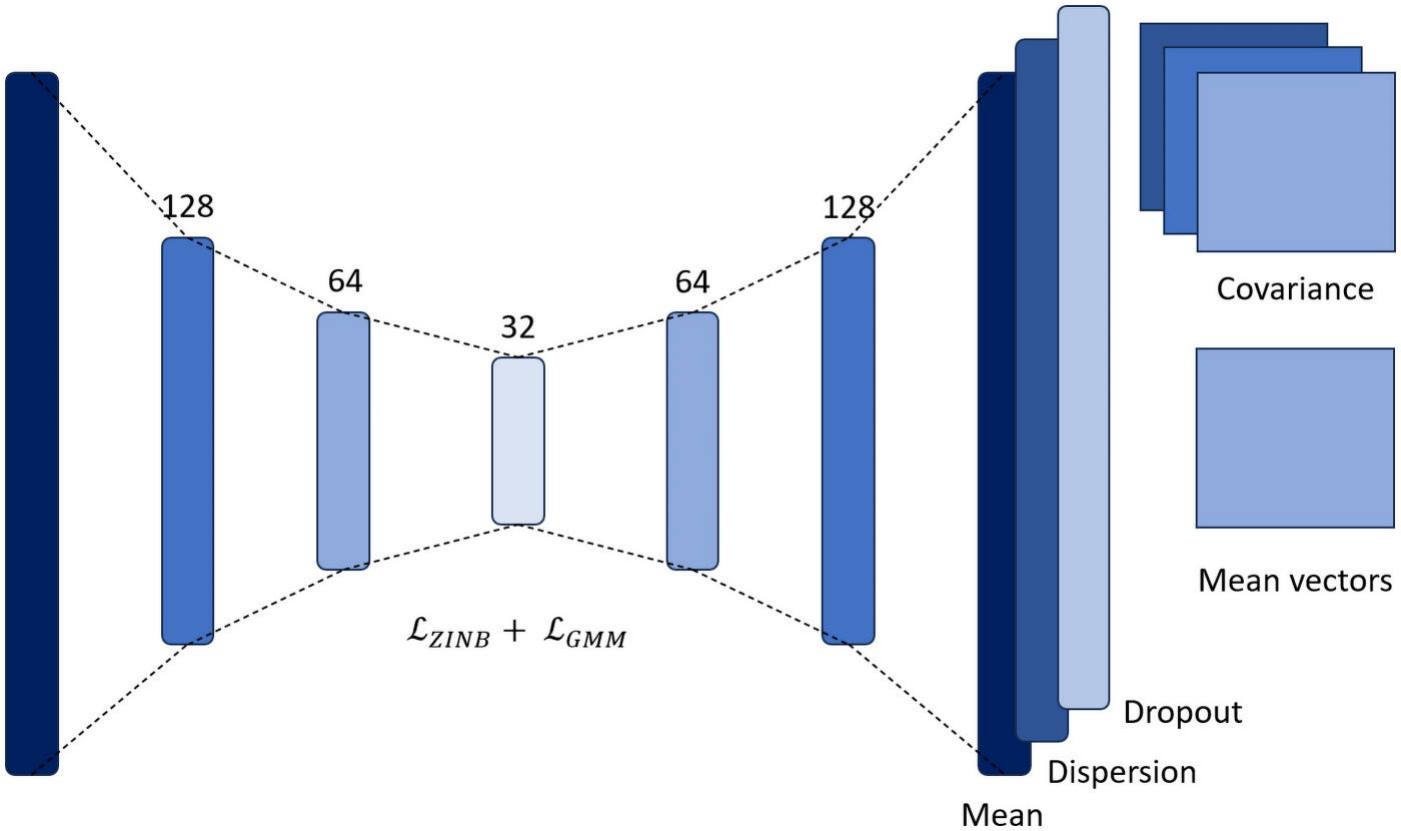
Neural network architecture. In the fine-tuning stage the model learns the parameters of each cluster.

In the second stage, fine-tuning is performed to cluster cells in the latent space using a Gaussian Mixture Model (GMM). The model assumes that the latent representation of each cluster *c* follows a Gaussian distribution with mean vector μ and covariance matrix Σ (Figure 2):


N(X μ,Σ)=12π|Σ|exp (−12(X−μ)tΣ−1(X−μ)).

The GMM-based loss is then defined as the negative log-likelihood over all *n* cells:


$$\begin{array}{cc} L_{\text{GMM}} & =\sum_{i=1}^{n}-ln\;p(x_{i}|\pi ,\mu ,\Sigma )\\ & =\sum_{i=1}^{n}-ln\sum_{c}\pi_{c}\cdot N(x_{i},\mu_{c},\Sigma_{c}). \end{array}$$

By incorporating this loss function, the model learns not only the mean, dispersion, and dropout as in DCA (Eraslan *et al.* 2019), but also the parameters that define the distributions of each data cluster (Figure 2). These data can be used to assign a probability to each point belonging to each cluster, also known as *soft clustering*. The hyperparameters were tested and selected following the DCA approach; further details can be found in the Supplementary table 1, available as supplementary data at *Bioinformatics Advances* online.

Some previous solutions based on autoencoders require as input the desired number of clusters. Taking into account that this value is not known in advance for many datasets, we implemented a procedure to predict the number of clusters. After pretraining, the *k*-MST algorithm is executed on the latent space and the number of clusters is retained for the training incorporating the GMM loss function.

## Methods

### Datasets for benchmark experiments

We used five publicly available scRNA-seq datasets obtained from various species, tissues, and sample sizes for further accuracy assessment of the proposed methods (Table 1). Additionally, we sequenced a new sample taken from the brain of a patient with refractory epilepsy during a scheduled surgery. For public datasets, the clusters identified in the original studies served as gold standard labels.

**Table 1:** Comparison of the datasets by species, tissue, number of cells, number of clusters and the percentage of zeros in the data.

Dataset	Species	Tissue	Cells	Clusters	% Zeros (%)
10X PBMC (Zheng *et al.* 2017)	Human	Blood	4340	8	96.08
Human Liver (MacParland *et al.* 2018)	Human	Liver	8444	11	90.76
Mouse bladder (Han *et al.* 2018)	Mouse	Bladder	2746	16	94.86
Macosko mouse retina (Macosko *et al.* 2015)	Mouse	Retina	14 653	39	88.34
Worm neuron (Cao *et al.* 2017)	Worm	Neuron	4186	10	98.61
Epilepsy	Human	Brain	3903	6	91.39

The epilepsy sample was selected from a cohort of patients treated by the Epilepsy Surgery service at Fundación Hospital de la Misericordia (HOMI) in Bogotá, Colombia. Patients diagnosed with refractory epilepsy, according to ILAE criteria, and identified as potential candidates for surgery by the pediatric epilepsy surgery group at HOMI were considered for inclusion. Exclusion criteria included autoimmune diseases, diabetes mellitus, and cancer. Participants who agreed to participate in the research study provided informed consent or assent. One patient with a diagnosis of right frontal structural and drug-resistant focal epilepsy was selected.

Brain tissue sample was collected from surgery, immediately transported to the laboratory on dry ice within 30 minutes of collection, and stored at −80°C until further processing. The frozen tissue was received and processed at the Princess Margaret Genomic Centre in Canada, according to the established protocols for nuclei extraction and purification of the sequencing center. The study employed the 10X Genomics 3’ v3.1 Nuc-Seq methodology, capturing roughly 7500 nuclei into droplets via the 10X Chromium system (3’ v3.1 kit). The library was designed to target an average read depth of 150,000 reads per nucleus. Mapping and feature counting were performed using STARsolo v2.7.11a (Kaminow et al., 2021) against the GRCh38-2020-A (Gencode v32/Ensembl 98) human reference genome. The count matrix was then imported into Seurat v5 (Hao *et al.* 2024) for preprocessing. Cells with abnormal mitochondrial content (major than 5%) and cells with fewer than 100 or more than 10,000 expressed genes were removed. Doublets were identified and removed from the dataset using scDblFinder v3.15 package (Germain *et al.* 2022).

To use this dataset for benchmarking purposes, a gold standard set of labels was constructed using the single cell resource published under the Human Protein Atlas Project (Karlsson *et al.* 2021; Siletti *et al.* 2023). This dataset comprises gene expression data across various human tissues and cell types, utilizing single-cell RNA sequencing (scRNA-seq), cell sorting, single-nuclei RNA sequencing (snRNA-seq), and bulk RNA-seq correlation analyses. It includes 557 individual cell type clusters representing 81 distinct cell types. Each cell was classified as neuronal (Inhibitory/GABAergic or Excitatory/Glutamatergic), non-neuronal (Astrocytes, Microglial cells, Oligodendrocyte precursor cells, Oligodendrocytes), or non-brain related cellular type (Others). In order to build an independent expression profile for each cell type, the average gene expression across replicates was calculated for each gene within each cell type of the HPA reference. For each cell, the Pearson correlation coefficient of its expression profile against each cell type was calculated. The cell was assigned to the cell type with the highest correlation coefficient. This procedure was tested in a publicly available dataset of 101 982 neuronal cells for which independent cell type assignments were performed (Pfisterer *et al.* 2020), achieving an assignment concordance of 0.93. Finally, cells assigned to non-brain cell types and cells with inconsistent locations in the t-SNE visualization were removed from the dataset.

### Preprocessing of the count matrices

We performed normalization in these experiments using the Scanpy package (Wolf *et al.* 2018), which normalizes each cell by total counts over all genes, so that every cell has the same total count after normalization. Size factors are computed for each cell, by dividing the count sum of the specific cell across all genes, divided by a constant factor *L*. In the predefined parameters of Scanpy, *L* is equal to the median raw count depth in the dataset. The normalization is then followed by a logarithmic scaling to complete the processing of the count matrix. In the Neural Network, an additional step was carried out following the normalization procedure outlined in scDCC (Tian *et al.* 2021). Specifically, a Standard Scaler was applied, ensuring that each cell has a mean of 0 and a standard deviation of 1.

For the graph-based algorithm, instead of performing a direct dimensionality reduction on the initial data, we selected the 5000 genes with the highest upward deviation from the adjusted variance. To achieve this, the variance $\sigma^{2}$ and mean μ for each gene in the log-normalized count matrix are calculated. Using these values and following the same idea as presented in (Amezquita *et al.* 2020), we fitted a curve of the form


$$\sigma^{2}=\frac{a\cdot \mu }{\mu^{n}+b},$$

finding the best possible values of *a* and *b*. The selected genes are the 5000 genes with the largest difference between the actual variance and the expected variance.

### Metrics

To evaluate the performance of the proposed models, we used three different supervised metrics. Specifically, we used accuracy, normalized mutual information (NMI), and the adjusted Rand index (ARI) to assess the degree of correspondence between the clusters identified by the algorithm and the true clusters.

### Tools

We compared the supervised metrics produced by our algorithms to those obtained using seven publicly available tools for scRNA-seq data clustering, three based on graphs, and four based on autoencoders. The tools based on graph clustering were Seurat (Hao *et al.* 2021), Phenograph (Levine *et al.* 2015) and SC3 (Kiselev *et al.* 2017). The tools based on autoencoders were scDCC (Tian *et al.* 2021), scDeepCluster (Tian *et al.* 2019), scCDCG (Xu et al., 2024) and scVAE (Gronbech *et al.* 2020). Each of these tools employs different clustering methodologies. Seurat uses a graph-based clustering algorithm on the *k*-NN graph of the cells in a lower-dimensional representation. SC3 utilizes a *k*-means algorithm combined with a consensus hierarchical clustering approach to determine the final clusters, requiring the number of clusters to be specified beforehand. Phenograph constructs a nearest-neighbor graph of cells, computes Jaccard similarities and partitions the graph into communities via the Louvain algorithm.

Among the autoencoder-based methods, scDeepCluster employs a deep autoencoder that models counts with a zero-inflated negative binomial distribution and incorporates a clustering-oriented loss based on Kullback–Leibler (KL) divergence (Tian *et al.* 2019). ScDCC extends this framework by further augmenting the loss function with pairwise constraints, encouraging well-separated latent representations (Tian *et al.* 2021). scCDCG combines an autoencoder, graph embeddings to capture cell–cell relationships, and an optimal-transport–based self-supervised objective that aligns latent representations with cluster assignments. Finally, scVAE uses a variational autoencoder to learn probabilistic latent embeddings, typically with negative binomial–family likelihoods.

For Seurat and SC3, we followed the procedures outlined in their tutorials using the default parameters. For scDeepCluster we ran the pytorch implementation available at https://github.com/ttgump/scDeepCluster_pytorch. This version implements a procedure to predict the number of clusters running the Louvain algorithm on the latent space representation after pretraining. Besides the number of clusters, the training process uses the cluster assignments and the centroids as prior information. In the case of scDCC, we had to make several modifications to the code in order to make it work with our current hardware architecture, and to fix bugs in the code. In particular, the script tried to read files that were either missing from the repository or unrelated to the scRNA-seq experiment data. Moreover, the software required a file with gold-standard clusters, making it impossible to execute for new datasets. The fixed version of this approach is available in the repository of scRANGE and it can be executed activating the option scDCC. We also performed fixes to the code of scCDCG because it had hard coded paths and specific parameters for the test datasets available with the distribution of this software. Moreover, the implementation also required true clusters as a mandatory parameter and it did not generate the predicted clusters as output. A detailed analysis of the code revealed that the procedure after pretraining requires a previously trained model, which by default is the model obtaining maximum accuracy during pre-training. Hence, this procedure can not be evaluated as an unsupervised clustering algorithm. Given this situation, for scCDCG we reported the performance metrics obtained at the last (100th) iteration of the pretraining process.

### Validation

**Figure 3:**
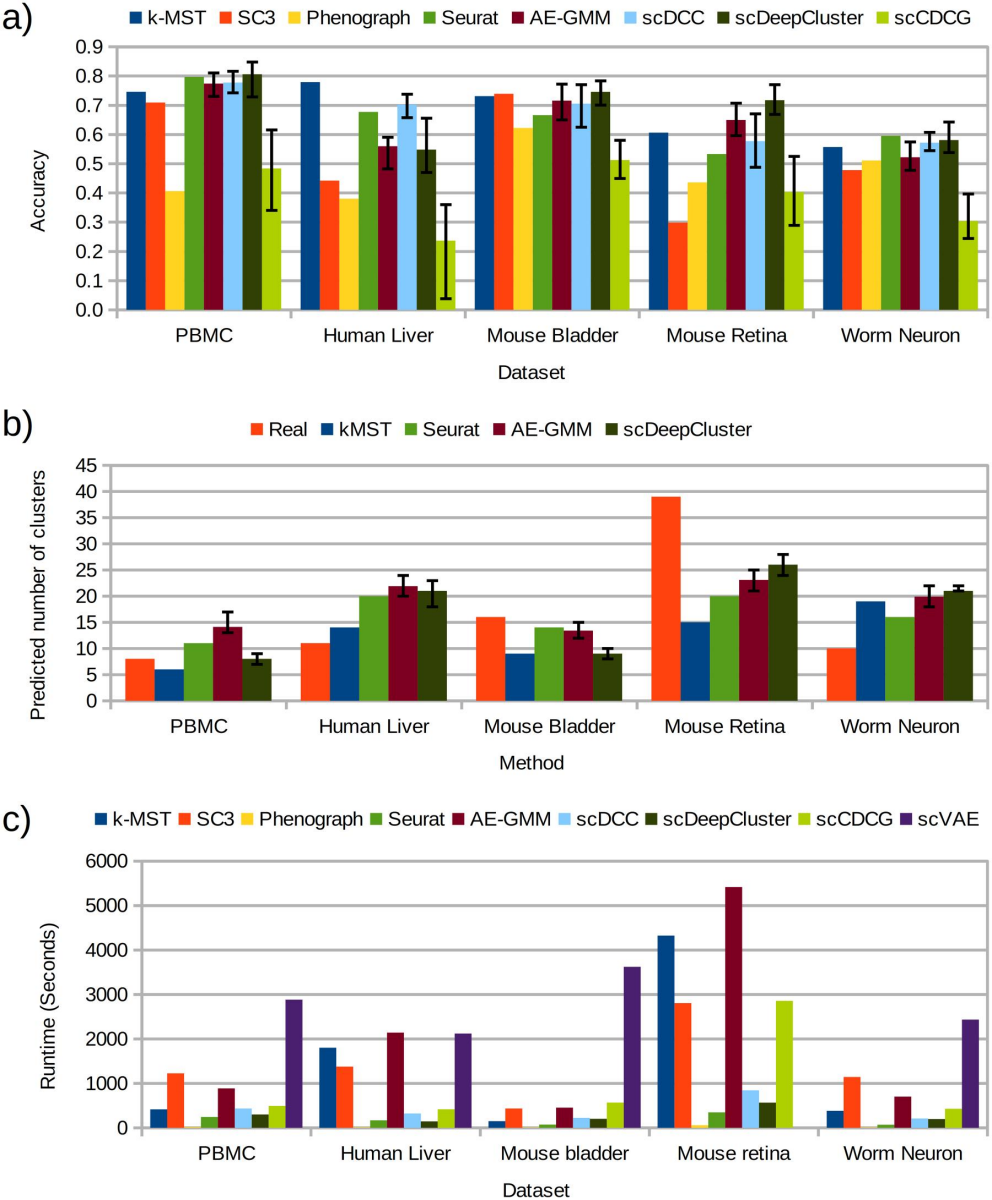
Performance comparison of different methods on open-source datasets. (a) Accuracy. (b) Number of predicted clusters. (c) Runtime. For methods based on neural networks the bar represents the average over 10 executions. Error bar limits represent the minimum and the maximum value.

Figure 3a shows a comparison of the accuracy values calculated from the clustering obtained running each tool on the publicly available datasets (data available in the supplementary table 2, available as supplementary data at *Bioinformatics Advances* online). Methods based on neural networks were executed ten times for each dataset using different random seeds to reduce the dependency of the results on weight initialization (Supplementary table 3, available as supplementary data at *Bioinformatics Advances* online). The figure shows the average over the executions in these cases. While the *k*-MST algorithm ranked first for the human liver dataset, scDeepCluster ranked first for the PBMC and the mouse datasets. Seurat ranked first for the worm neuron dataset.

One important determinant of the accuracy of each method is the prediction of the number of clusters. Taking into account that for many real datasets the number of clusters is not known in advance, both methods presented in this manuscript estimate the number of clusters. Figure 3b shows a comparison between the number of clusters reported by the *k*-MST, Seurat, AE-GMM and scDeepCluster, compared to the number of clusters considered as gold-standard for each dataset. All methods consistently underestimated the number of clusters in the mouse datasets (which have the largest expected numbers), and overestimated the number of clusters in the Human liver and Worm neuron datasets. The scDeepCluster method predicted the correct number of clusters for the PBMC dataset in five runs. This partly explained why no method achieved accuracies larger than 0.85 for any dataset. While scDeepCluster and Seurat achieved the predictions closest to the real number in two of the five datasets, the prediction of *k*-MST ranked first for the human liver dataset, partly explaining the superior performance of *k*-MST in this dataset. In order to make a fair comparison, methods that require the number of clusters as input (SC3, scDCC and scCDCG, and scVAE) were executed with the number of clusters predicted by the *k*-MST algorithm.

Among the graph-based methods, SC3 outperformed the *k*-MST algorithm in one dataset, but it showed a poor performance in the datasets with the lowest percentage of zero entries (Human liver and Mouse retina). Phenograph ranked last in three datasets and second to last in the other two datasets. Seurat showed competitive results ranking first among all methods for the PBMC and the worm neuron datasets. However, its accuracy was between 0.05 and 0.1 lower than the accuracy of the *k*-MST in the other three datasets. In contrast, *k*-MST had a more stable performance, ranking first among graph-based methods in two datasets and ranking second for the other datasets, with accuracy differences lower than 0.05 against other graph based methods.

We performed additional experiments to assess the behavior of different preprocessing alternatives and different parameter settings for the *k*-MST method. The default preprocessing includes the selection of the 5000 top genes according to the mean-variance ratio (see the section “Preprocessing of the count matrices” for details). We compared the following alternatives: (a) select the 5000 genes with highest variance; (b) Correct dropouts before using the mean-variance filter; (c) Perform error correction but do not filter genes; (d) Skip error correction and gene filtering; and (e) Perform a principal component analysis (PCA) and calculate correlations on the latent space (Supplementary table 2, available as supplementary data at *Bioinformatics Advances* online). For error correction, we used the method proposed in (Eraslan *et al.* 2019) where an autoencoder with ZINB loss was employed to address dropouts. The output of this neural network was treated as a corrected dataset. None of the alternatives consistently improved the quality of the results across datasets, but three alternatives achieved improved accuracy (compared to the mean-variance filter) for three different datasets, respectively. Performing error correction before the mean-variance filtering raised the accuracy to 0.662 for the mouse retina dataset. Conversely, the alternative skipping error correction and filtering achieved an accuracy of 0.611 for the worm neuron dataset. Applying PCA before the graph construction achieved a modest increase in accuracy for the Mouse bladder dataset.

We also explored the impact of varying two parameters of the *k*-MST approach: the number of genes selected with the mean–variance strategy and the value of *k* used for constructing the graph (Supplementary Table 4, available as supplementary data at *Bioinformatics Advances* online). The effect of gene selection was dataset-specific. In the Macosko mouse retina dataset, increasing the number of selected genes worsened accuracy, whereas in the Human Liver and Human Brain datasets, including more genes produced higher accuracy. A similar dataset dependency was observed for the *k* parameter (number of MSTs computed). In the Worm Neuron dataset, higher *k* values led to more accurate and stable clustering, while in other datasets the effect was less consistent and did not follow a clear trend.

Among the neural network methods, the AE-GMM showed a significantly better accuracy than scCDCG in all datasets (*p*-value < 0.001 of a Wilcoxon rank test). Compared to scDCC, the average accuracy of AE-GMM was 0.07 larger for the mouse retina dataset. However, the *p*-value was 0.014, because scDCC had a larger standard deviation across replicates than AE-GMM (0.06 versus 0.03). In contrast, scDCC achieved significantly better accuracy than AE-GMM for the human liver and the worm neuron datasets. Compared to scDeepCluster, AE-GMM had a lower average accuracy in four datasets. The difference was significant for the mouse retina (*p = *0.002) and the Worm neuron (*p = *0.0008) datasets. The scVAE method could not be included in this comparison because it showed a high computational cost and it failed for the Mouse Retina dataset, requiring more than 128 GB of RAM.

The Mouse Retina dataset contains more than 14 000 cells and over 30 distinct clusters. We investigated in detail the training dynamics of AE-GMM for this dataset, taking into account that it achieved an improvement of 0.05 in accuracy, compared to scDCC. The accuracy across epochs for 10 independent runs during the fine-tuning stage showed a consistent increase in accuracy (Supplementary figure 1, available as supplementary data at *Bioinformatics Advances* online). Despite the complexity of this dataset, AE-GMM consistently improved across runs, stabilizing after approximately 60 epochs. These results illustrate how the stabilization of clusters under the GMM formulation can improve clustering performance in highly complex datasets.

We also assessed the performance of the evaluated methods using the alternative measurements NMI and ARI (Supplementary figure 2 and Supplementary tables 2 and 3, available as supplementary data at *Bioinformatics Advances* online). While NMI compares clusters based on cross entropy, ARI focuses the comparison on pairs of elements. Hence, a-priori these metrics should be less sensitive to differences in the number of clusters, compared to accuracy. The NMI values were consistently larger than the accuracy values (0.06 on average). Regarding changes in rankings (particularly in top performers), in the mouse bladder dataset scDeepCluster went down from the first to the third position, below AE-GMM and Seurat, and tied with *k*-MST. Seurat had lower NMI than scDeepCluster, scDCC, *k*-MST and AE-GMM in the worm neuron dataset. Regarding ARI, the ARI values were the lowest among the three metrics, showing an average difference of 0.08 against the accuracy values. Seurat outperformed the *k*-MST for the human liver dataset, but the *k*-MST outperformed scDeepCluster and SC3 for the mouse bladder dataset and Seurat for the worm neuron dataset. scDeepCluster had better ARI than Seurat and *k*-MST in the latter dataset, becoming the top performer according to this metric.

Figure 3c shows a comparison of the different tools in terms of computational efficiency. The tool scVAE consistently ranked last in this metric, taking more than 2000 seconds for all datasets. As mentioned above, we were not able to run this tool for the mouse retina dataset. Consistent with the analysis of computational complexity, the *k*-MST method took more time than Seurat and Phenograph. Particularly for the complex mouse retina dataset it took about 1.5 hours. Given that the AE-GMM uses the *k*-MST method on the latent space to infer the number of clusters, the AE-GMM takes a runtime longer than the *k*-MST, matching the runtime taken by scVAE for the human liver dataset. Phenograph, Seurat, scDeepCluster and scDCC ranked first, second, third and fourth respectively.

#### A new dataset for benchmarking of scRNA-seq cell clustering methods

**Figure 4:**
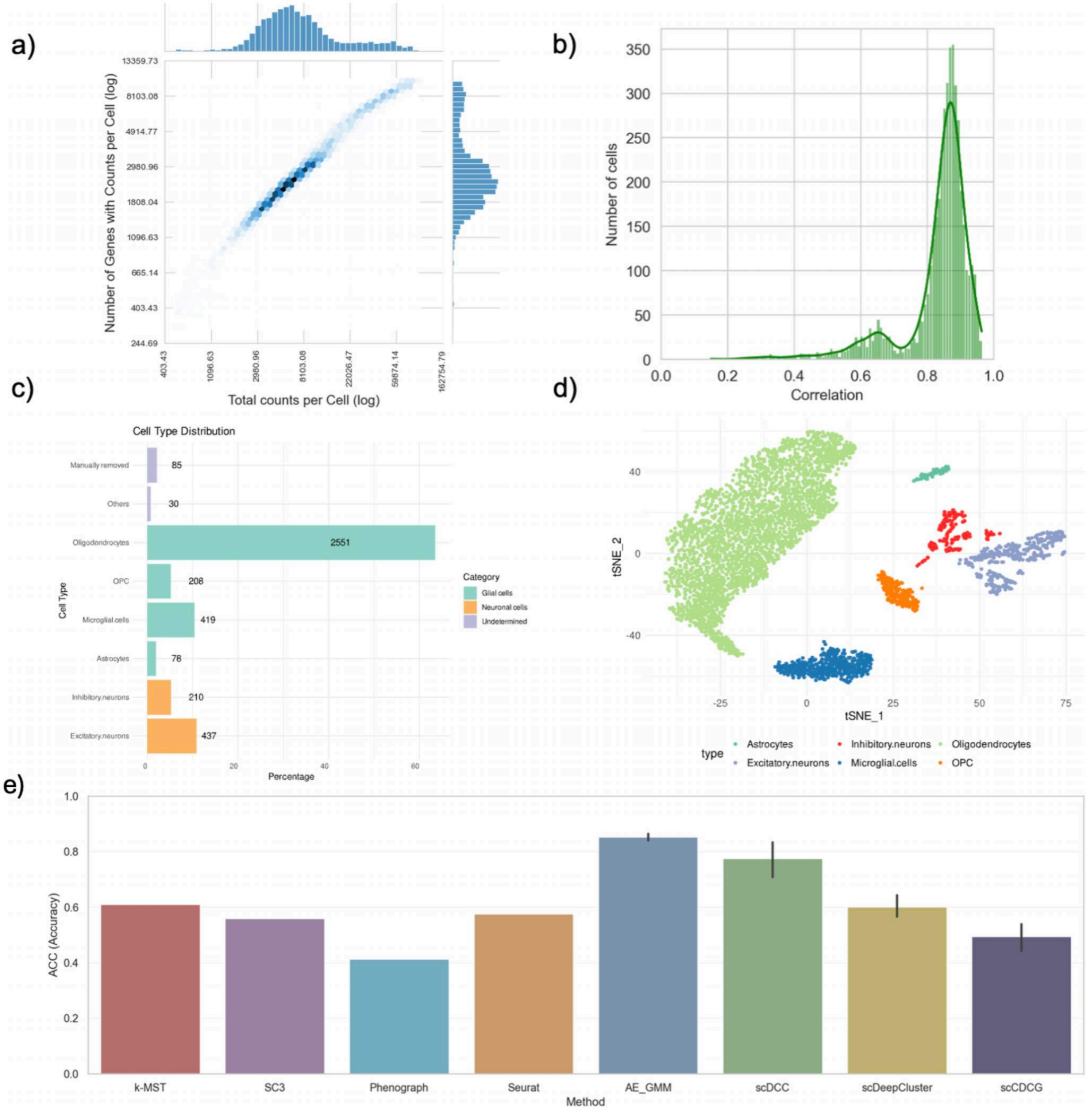
(a) Sequencing depth of the sample. (b) Histogram of maximum correlation values between cells and clusters defined at the human protein Atlas. (c) Percentage values of cell types presented in Epilepsy dataset. The count of cells for each type is indicated. (d) t-SNE visualization of cell types used as the epilepsy gold standard dataset. (e) Supervised metrics for the epilepsy dataset among the different methodologies. For methods based on neural networks the bar represents the average over 10 executions. Error bar limits represent the minimum and the maximum value.

Single nuclei RNA sequencing of the epilepsy patient resulted in a total of $1,137\times 10^{6}$ reads. From these, 97.4% mapped to the human reference genome with 70.2% of reads mapping uniquely to an intronic region. The raw count matrix had a total of 91.29% zero values before gene filtering. After filtering cells with abnormal mitochondrial content, cells with an aberrant number of expressed genes, and doublets the dataset included a total of 4,018 high-quality cells having expected values for the number of molecules per cell, the number of genes per cell and the mitochondrial gene content for cells. The final distribution of counts per cell and sequenced genes are presented in Figure 4a.

Correlations between the gene profiles of the sequenced cells and the Human Protein Atlas ((Uhlén *et al.* 2015)) database were calculated to perform the supervised assignment method described above. Figure 4b shows that the maximum correlations were distributed between 0.7 and 1 for most cells, with a mean value of 0.83. As shown in Figure 5c, the dataset was dominated by Oligodendrocytes, representing 63.5% (*n = *2551) of the cells, followed by Excitatory cells at 10.9% (*n = *437), and Microglial cells at 10.4% (*n = *419). With less than 300 representatives, the Inhibitory Neurons represent 5.22% (*n = *210), followed by oligodendrociyte Precursor Cells (OPC) with 5.17% (*n = *208), and a final percentage of 1.94% (*n = *78) for Astrocytes. Figure 5d shows a t-SNE visualization of the annotated cells. Based on this analysis, 85 probably misclassified cells (42 neurons, and 43 glial cells) were removed from the dataset for benchmarking purposes. A final number of 3903 annotated cells were used as the gold standard after removing the “Others” category.

After assigning cell types based on the Human Protein Atlas database, we executed the proposed algorithms and we used the assigned clusters to compute the three supervised metrics. Figure 4 shows that AE-GMM achieved the highest accuracy with an average 0.853. Similar to the case with the mouse retina dataset, the difference between AE-GMM and scDCC (0.08) was not evaluated as significant (*p*-value = 0.13) because the standard deviation of scDCC (0.12) was much larger than that observed for AE-GMM (0.02). In contrast with the results observed with the previously analyzed datasets, the accuracy of AE-GMM in this case was significantly larger than that achieved by scDeepCluster (0.6, *p = *0.0002). For this dataset all graph-based methods ranked below the AE-GMM with accuracies around 0.6. In this case, the rankings were mostly stable across the three metrics (Supplementary table 2, available as supplementary data at *Bioinformatics Advances* online). The *k*-MST ranked first among graph based methods for accuracy and NMI, closely followed by Seurat, which ranked first for ARI.

Supplementary Figure 3, available as supplementary data at *Bioinformatics Advances* online presents the t-SNE representation of the clustering techniques applied to the epilepsy dataset. The clustering methods SC3, *k*-MST, and Seurat exhibit overclustering within the oligodendrocyte cells, dividing it into two or three subclusters. Adjusting the Seurat results by merging clusters 0 and 1 leads to enhanced clustering performance, with an accuracy of 0.88, ARI of 0.97, and NMI of 0.89. For *k*-MST, merging clusters 0, 2, and 10 results in an accuracy of 0.94, NMI of 0.93, and ARI of 0.97. Similarly, combining clusters 1 and 2 for SC3 yields an accuracy of 0.92, NMI of 0.92, and ARI of 0.96. These findings indicate that these methods could benefit from post-processing adjustments to correct for overclustering.

## Discussion

Identification of cell types from scRNA-seq data is an interesting bioinformatics problem, due to the high dimensionality and non linearity of the data. From the computer science perspective, the problem is an instance of the general problem of clustering high dimensional data, for which a wide variety of algorithms have been proposed up to date (Raha *et al.* 2025). Given the wide use of scRNA in different experimental settings in a large number of species, software implementing accurate algorithms for unsupervised cell clustering of scRNA data is likely to have a large number of users. In this work we contributed two algorithmical improvements building upon two major strategies for scRNA cell clustering (graph based and autoencoders). Experiments with a large number of datasets show that none of the methods is inherently superior. However, the proposed methods achieve a competitive and stable performance across experiments and metrics. Both methods are implemented in the software package scRANGE, to facilitate independent reproduction of the results and to facilitate the use of these methods by different research groups. Our experience with current methods for cell clustering in scRNA data suggests that implementation using basic software engineering practices is important to translate good algorithmic ideas to useful solutions. Although a large number of algorithmic approaches for clustering single-cell data have been published in recent years, Seurat is a current standard de-facto for the analysis of these data, primarily due to the usability of the tool. Conversely, the potential improvement in accuracy that could be achieved using models such as that implemented in scDCC can not be realized due to software bugs and issues with version management.

One main outcome of the benchmarking process is that the estimation of number of clusters has an important effect on the calculation of the comparison metrics. Particularly, the accuracy metric can be reduced if the number of clusters predicted by the method is not consistent with the number of clusters of the gold-standard (supplementary table 3, available as supplementary data at *Bioinformatics Advances* online). This was not considered an issue for some recent benchmark experiments because the methods were executed providing as a parameter a number of clusters consistent with those of the gold-standards (Tian *et al.* 2021). The two methods presented in this manuscript include a procedure to estimate the number of clusters as part of the algorithm. In the case of the k-MST method, the estimation is a byproduct of the community detection step running the Louvain algorithm on the k-MST graph. For AE-GMM, the algorithm runs k-MST from the latent space of the autoencoder after pretraining. The experiments with real datasets including both methods and the methods implemented in Seurat and scDeepCluster indicate that no method was able to predict the exact number of clusters. Considering that the real number of clusters is usually not known in advance, this result suggests that future work could be specifically directed to address the estimation of number of clusters. One difficulty related to this goal is that the number of gold-standard clusters in the benchmark datasets is subjective to the judgment of the researchers, and it can be biased by the methods originally used to analyze the testing data. Even in the epilepsy dataset, the expected number of clusters corresponds to the major brain cell types. This penalizes methods that could be producing possible correct finer clusters. The estimation procedures implemented in our algorithms can be good starting points to address this situation and, from the user perspective, they can be considered interesting alternatives for exploratory analysis of new datasets.

The *k*-MST algorithm proposed in this article is a graph-based clustering method in which successive minimum spanning trees are calculated and aggregated to generate the final structure. This construction is fundamentally different than the *k*-NN graph implemented in Seurat (Hao *et al.* 2021), which is built just taking the *k* nearest neighbors of each node. As a consequence, in both graphs each node has at least *k* neighbors, but the *k*-MST allows more edges per node for nodes with a large number of low cost connections. We believe that this helps to capture more relational information between cells, improving the outcome of the subsequent clustering using the Louvain algorithm. According to the benchmark experiments, the *k*-MST algorithm provides improved accuracy compared to Seurat in four out of six analyzed datasets. However, testing with a larger number of benchmark datasets is needed to provide a definite ranking between these algorithms. Another aspect that could explain the difference in accuracy is the preprocessing for dimensionality reduction. The *k*-NN graph is usually built after a reduction of dimensionality using a technique such as PCA. Instead, we built the *k*-MST graph from correlations calculated directly from the counts matrix without dimensionality reduction. Although the graph could also be built from a PCA latent space, our experiments indicate that the accuracy improves if raw counts are used as source to calculate correlations (Supplementary table 2, available as supplementary data at *Bioinformatics Advances* online).

The second proposed algorithm, AE-GMM, is a deep learning-based method. Unlike traditional autoencoder approaches, this algorithm integrates clustering directly into the training process, learning the parameters that characterize each cluster. Specifically, the network is first pretrained using a ZINB loss, as in DCA, to account for overdispersion and dropout effects, and is then fine-tuned through a GMM loss that estimates the parameters $\left(\pi_{c},\mu_{c},\Sigma_{c}\right)$ of each cluster in the latent space. The inclusion of the GMM loss allows the resulting clusters to have more flexible shapes, such as ellipsoids, in contrast to methods like *k*-Means, which impose stricter constraints on cluster shapes. This flexibility allowed the algorithm to obtain the highest average values of all metrics for the epilepsy dataset. An accuracy improvement performing fine tuning with the GMM loss function was evident in the mouse retina dataset. The accuracy consistently increased from around 0.5 up to 0.7 across different runs. Beyond reliability, a main advantage of our solution is related to the improvements in the implementation that we performed to provide users a better experience running the software. Given the difficulties that we experienced running the available version of scDCC, we implemented an option in scRANGE to run the scDCC model from our software. We expect that this facilitates future users and method developers to run this model along with the AE-GMM method.

Regarding computational efficiency, the *k*-MST algorithm was less efficient than other graph based methods, including Seurat. This could be explained in part because correlations are calculated directly from the raw matrix without dimensionality reduction, and also by the theoretical time complexity of the algorithm. Although this is a comparative disadvantage of the method, in practice the *k*-MST could cluster tens of thousands of cells within a couple of hours. Future works include investigating alternatives to improve the computational efficiency of this method. The AE-GMM had a larger runtime compared to other autoencoder methods because it predicts the number of clusters running the *k*-MST method on the latent space. Although the current methods based on autoencoders achieved a low runtime, it is worth clarifying that methods based on neural networks only achieve this efficiency if they are executed on GPU infrastructures. This can be considered as a disadvantage because not all labs have the hardware and technical skills required to operate software in this infrastructure.

Finally, this article presents a new single-cell RNA-seq dataset obtained from a patient with refractory epilepsy. Thanks to the decreasing costs of sequencing, this dataset was sequenced with a depth per cell higher than that obtained in previous datasets used for benchmarking of scRNA-seq clustering methods. This translated into a relatively low percentage of zero entries. We believe that this approach will be taken by different groups currently designing scRNA-seq experiments, making this dataset a valuable contribution to increase the diversity of scRNA-seq datasets for benchmarking of current and new methodologies developed by different research groups. We followed a supervised approach based on comparisons against the human protein atlas to build the gold standard cell annotations for this dataset. Although we acknowledge that this method could produce some erroneous assignments, the proposed method is orthogonal to the unsupervised clustering methods. Hence, errors should not be biased to favor any particular clustering algorithm. The performance of the autoencoders implemented in AE-GMM and in scDCC was noticeably good for the epilepsy dataset, compared to the performance of the graph based methods. This result suggests that architecture of the autoencoders is able to improve its performance taking advantage of the additional information provided by datasets with a lower percentage of zero entries. We believe that this dataset can contribute to increase the heterogeneity of benchmark datasets, providing a useful resource for developers of scRNA-seq data analysis methods.

## Supplementary Material

vbag121_Supplementary_Data

## Data Availability

The algorithms and the datasets supporting the results presented in this article are available as part of the distribution of scRANGE: https://github.com/mvrobles/scRANGE. The matrix count file in MTX format for the epilepsy patient has been deposited in the NCBI Gene Expression Omnibus (GEO) at https://www.ncbi.nlm.nih.gov/geo/query/acc.cgi, and it can be accessed with the accession number GSM9101271.
