# Supplementary material for: New algorithms for unsupervised cell clustering from scRNA-seq data
Source: Bioinform Adv. 2026 Apr 29;6(1):vbag121. doi: 10.1093/bioadv/vbag121 (PMC13176612; doi:10.1093/bioadv/vbag121)

Supplementary Figure 1. Accuracy values during the fine-tuning stage of AE-GMM on the Mouse Retina dataset. Results are shown across 100 epochs for 10 independent runs with different random seeds.

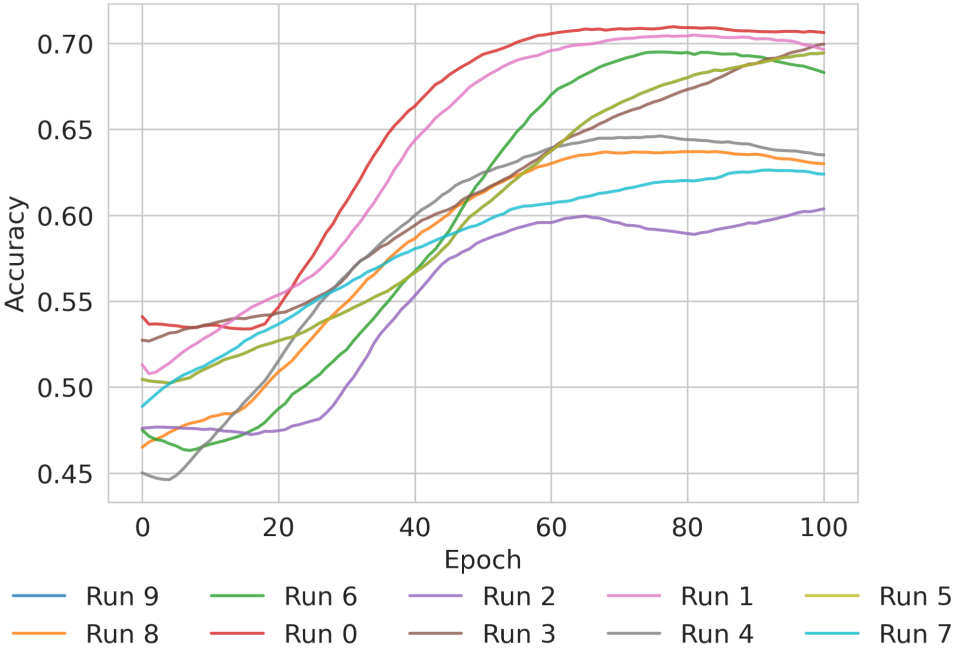

Supplementary Figure 2. Performance comparison of different methods based on autoencoders for clustering of open-source datasets. (a) NMI (b) ARI. For methods based on neural networks the bar represents the average over 10 executions. Error bar limits represent the minimum and the maximum value.

a)

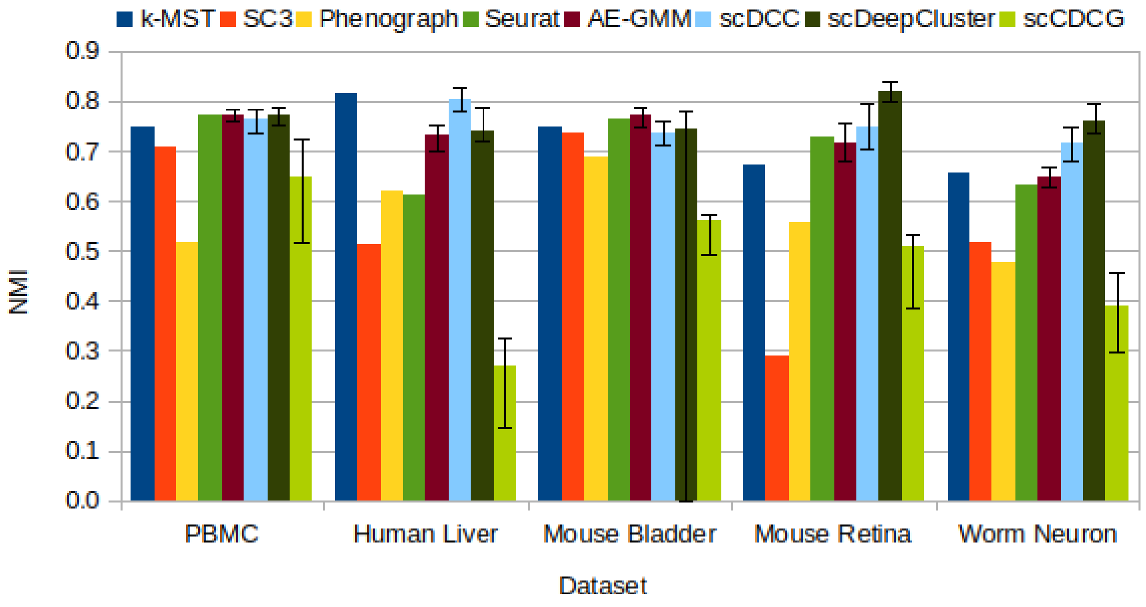

b)

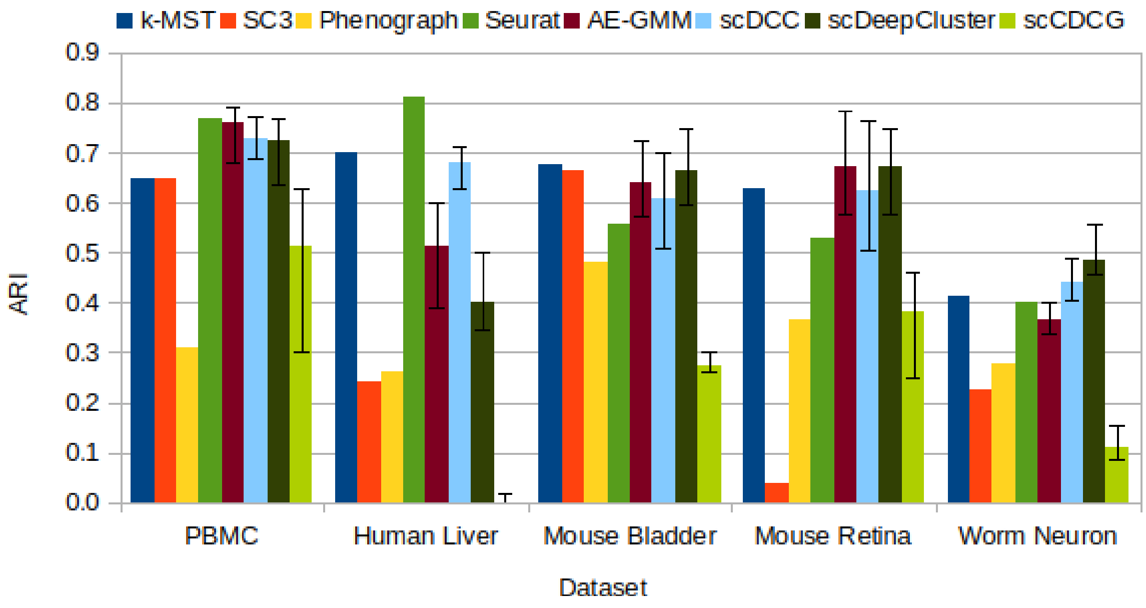

Supplementary Figure 3. t-SNE representation of the results of different clustering techniques for the epilepsy dataset.

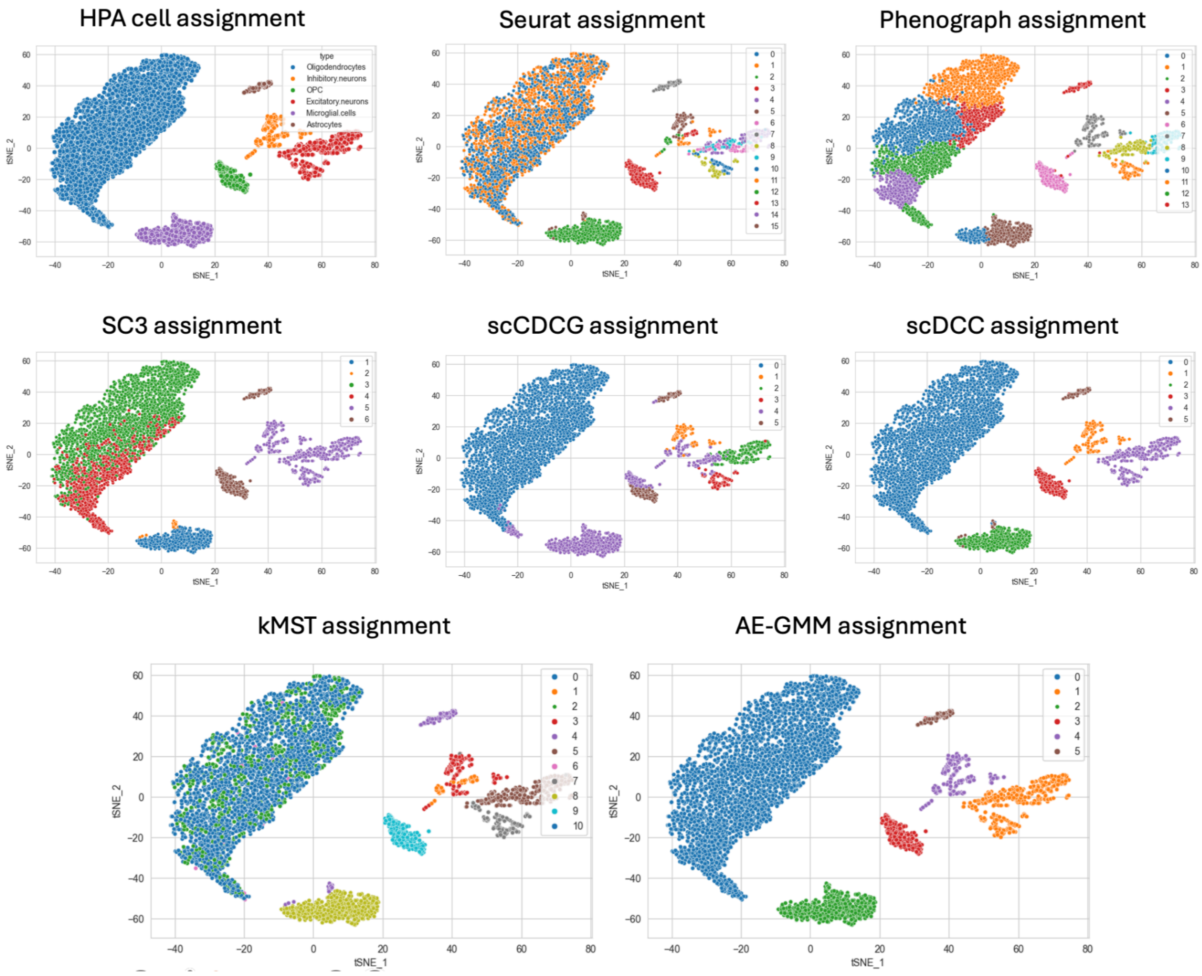

Supplement: vbag121_Supplementary_Data [file vbag121_supplementary_data.zip › SupplementaryFigures_20260404.pdf]
